# Supplementary figures and images for: Spatial Organization of the Gene Regulatory Program: An Information Theoretical Approach to Breast Cancer Transcriptomics
Source: Entropy (Basel). 2019 Feb 19;21(2):195. doi: 10.3390/e21020195 (PMC7514677; doi:10.3390/e21020195)

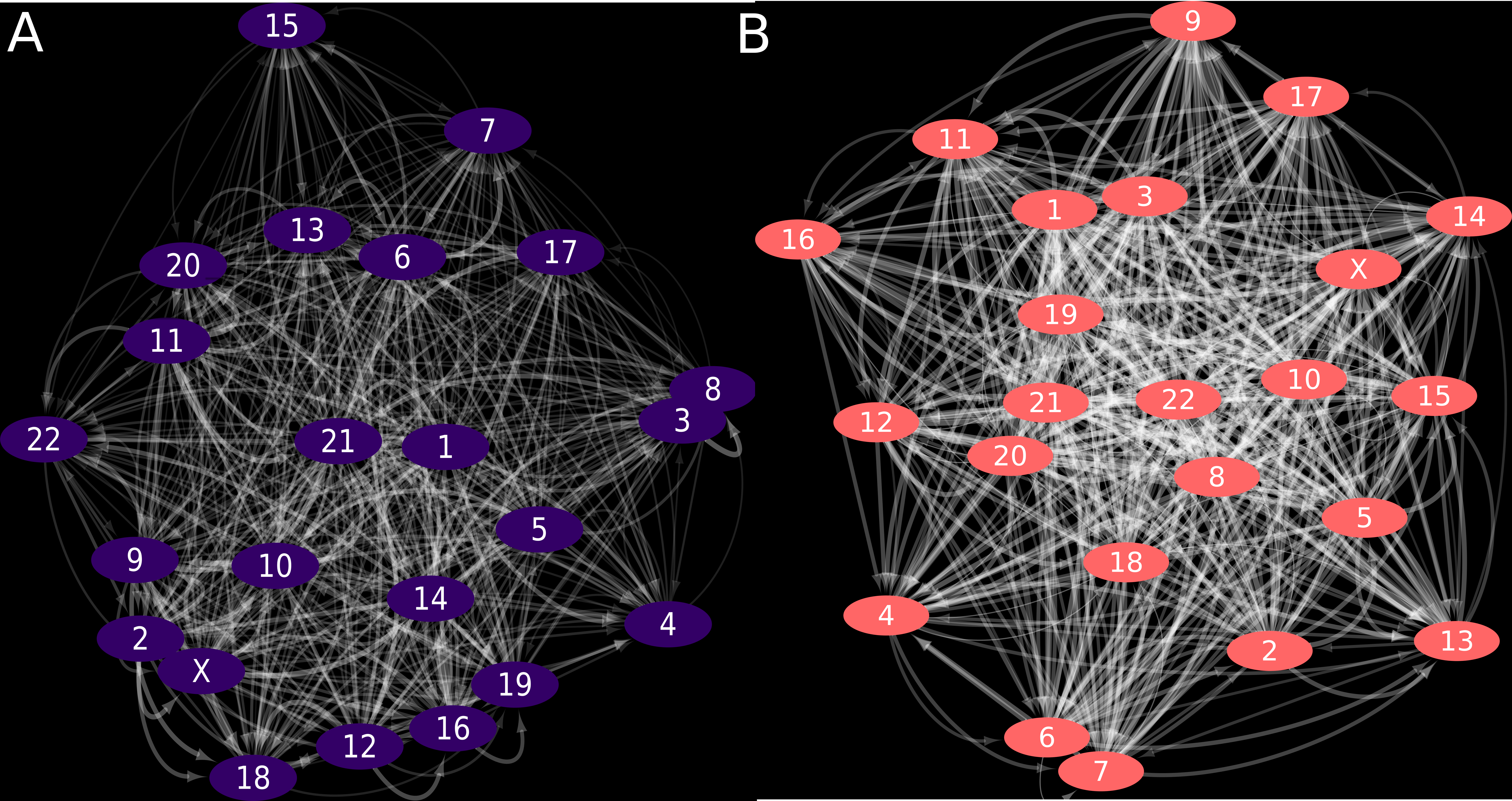

Supplement: Supplementary file 1 [file entropy-21-00195-s001.zip › Supplementary/SupplementaryFile02.png]

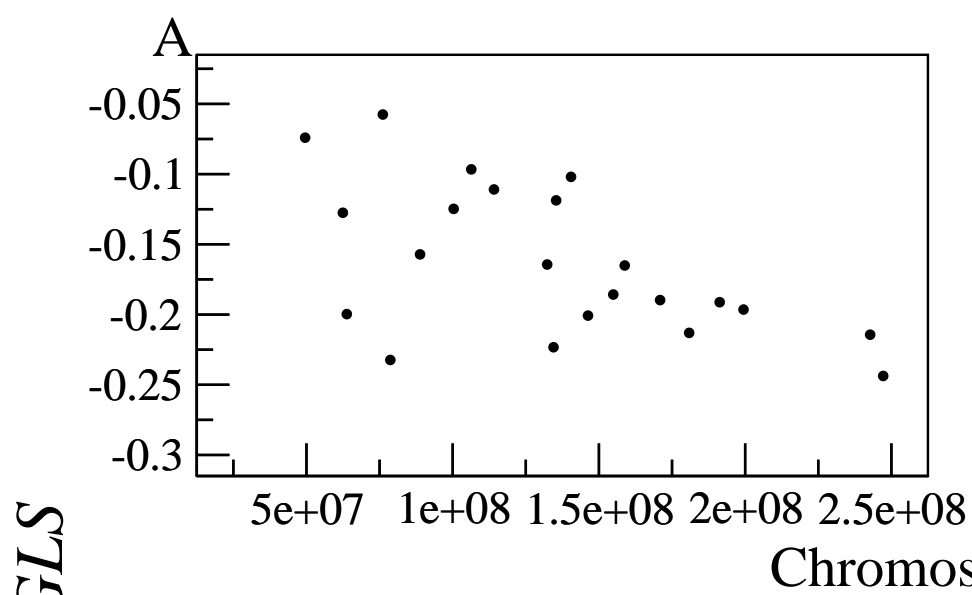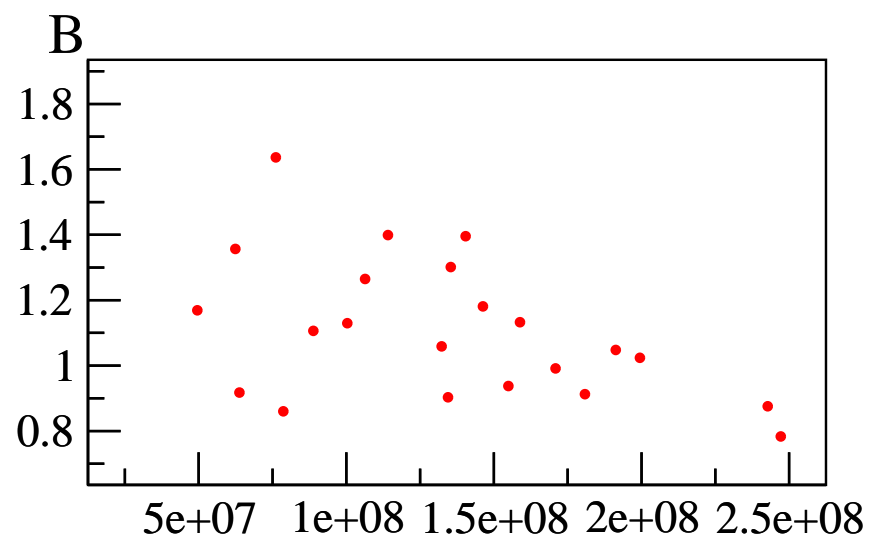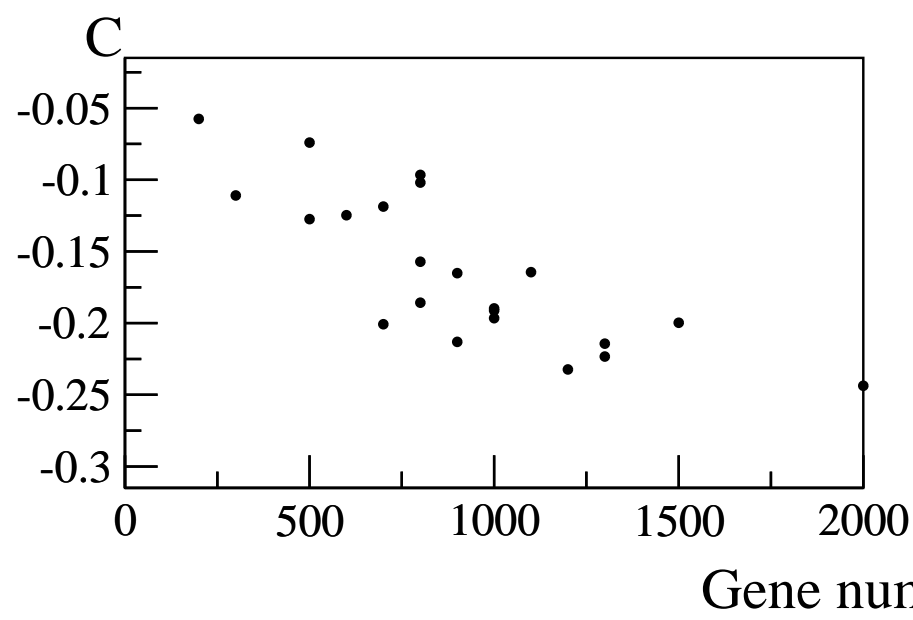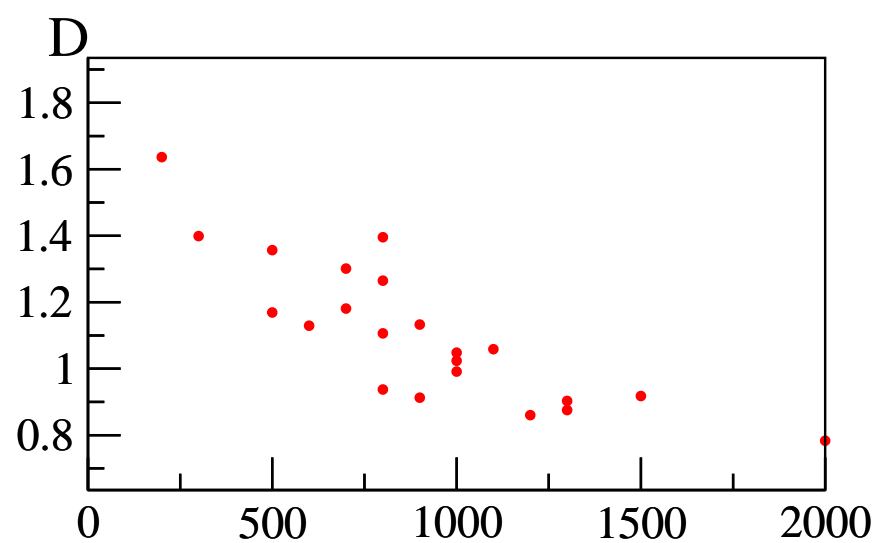

*GLR*

Supplement: Supplementary file 1 [file entropy-21-00195-s001.zip › Supplementary/Supplementary Fig3.pdf]
